# Strip1 Is a Novel Negative Regulator of Cardiomyocyte Hypertrophy

**DOI:** 10.3390/cells15060540

**Published:** 2026-03-18

**Authors:** Emanuel Heilein, Lucia Sophie Kilian, Samuel Sossalla, Benjamin Meder, Mirko Völkers, Karen S. Frese, Sabine Herch, Norbert Frey, Matthias Eden

**Affiliations:** 1 Department of Internal Medicine III, University of Heidelberg, 69120 Heidelberg, Germany; emanuel@heilein.de (E.H.); mirko.voelkers@med.uni-heidelberg.de (M.V.); karen.frese@med.uniheidelberg.de (K.S.F.); sabine.herch@med.uni-heidelberg.de (S.H.); norbert.frey@med.uni-heidelberg.de (N.F.); 2 German Centre for Cardiovascular Research, 69120 Heidelberg, Germany; 3 Department of Internal Medicine III (Cardiology and Angiology), University Hospital Schleswig-Holstein, 24105 Kiel, Germany; 4 Department of Cardiology, Giessen and Kerckhoff Heart and Lung Centre, University of Giessen, 61231 Bad Nauheim, Germany

**Keywords:** strip1, heart failure, cardiac hypertrophy, cardiomyopathy, STRIPAK, NRVCM, nucleolus, in vitro, cardiac signaling

## Abstract

Pathological cardiac hypertrophy is a critical factor leading to cardiomyopathy and ultimately heart failure. While several signaling pathways controlling cardiac hypertrophy have been identified, the molecular mechanisms underlying their precise regulation remain incompletely understood. Strip1, a structural component of STRIPAK complexes, has been implicated in various cellular functions; however, its role in cardiomyocytes is uncharacterized. Here we identify Strip1 as a potent anti-hypertrophic factor, controlling cell size and the hypertrophic gene program in neonatal rat ventricular cardiomyocytes (NRVCMs). STRIP1 expression was found to be significantly reduced in human dilated and ischemic cardiomyopathies (DCM/ICM), as well as in murine stress model induced by transverse aortic constriction (TAC). In a knockdown model with morpholino-driven STRIP1 reduction in zebrafish in vivo, impaired cardiac function and heart failure–like features were observed. Interestingly, Strip1 localized to the nucleolus in NRVCMs, suggesting a putative nuclear/epigenetic role in cardiomyocytes. Furthermore, our data support association of Strip1 with cardiac STRIPAK complex, modulating kinase activities, including MST1/MST2 and MST4. Mechanistically, Strip1 appears to influence prohypertrophic signaling, including Hippo- and Calcineurin/NFAT-related pathways, which may contribute to pathological cardiac remodeling. Collectively, these findings establish Strip1 as an important modulator of cardiomyocyte hypertrophy and a potential therapeutic target for cardiomyopathy and heart failure.

## 1. Introduction

Cardiac hypertrophy represents a fundamental adaptive response of the heart to increased workload, aimed at maintaining function and reducing ventricular wall stress [1]. Physiological hypertrophy, as observed, e.g., in athletes, is beneficial and reversible [2]. In contrast, pathological hypertrophy, triggered by chronic pressure overload, is a process distinct in both morphology and molecular profile that often leads to heart failure and thus poses a major risk for cardiovascular morbidity and mortality [1,2]. It is driven by a complex network of intracellular signaling pathways that remain incompletely understood, highlighting the need to identify novel regulatory pathways that govern this pathological progression [2,3].

Striatin-interacting phosphatase and kinase (STRIPAK) complexes represent evolutionarily highly conserved multiprotein assemblies that have gained attention as critical regulators of numerous physiological and pathological processes, including tumorgenesis, embryonic neural development, and vascular formation [4,5,6]. Recent evidence has increasingly highlighted their importance in cardiac function, particularly in relation to hypertrophy and heart failure [6]. Initial insights into the role of STRIPAK in cardiac function emerged from studies on its components, PP2A, striatins, and SLMAP [7,8,9]. Protein phosphatase 2A (PP2A), which acts within the STRIPAK complex by restraining kinase activity [6], has been identified as a key negative regulator of pathological cardiac remodeling, as transgenic mice overexpressing PP2A are protected from isoproterenol-induced cardiac hypertrophy [9]. Furthermore, heterozygous gene deletion of Striatins in mice identified them as inhibitors of angiotensin II-induced cardiac hypertrophy, while SLMAP was shown to alter the structure of the sarcomeric reticulum and calcium uptake, thereby regulating cardiac contractility [7,8].

We recently identified a STRIPAK member, MST4, as a novel cardiac kinase with potential relevance in heart disease. While its role in cardiomyocytes had not been previously explored, we found MST4 to be highly enriched in rodent hearts and markedly upregulated in human samples from patients with DCM. In NRVCMs, MST4 interacts with STRIPAK components and localizes to the intercalated disc. Its overexpression induces cardiomyocyte hypertrophy, enhances contractility, and reduces apoptosis. Phosphoproteomic analyses revealed potential targets at the intercalated disc, supporting a role of MST4 in regulating cardiomyocyte growth and survival in the context of cardiac remodeling [10]. In addition to its role within STRIPAK complexes, MST4 has been shown to phosphorylate the cytoskeletal protein Ezrin at Thr567, associated with changes in actin organization, which is used as a readout of MST4 kinase activity [11].

Previously we also discovered striatin-interacting protein 2 (Strip2), which we termed “myocardium-enriched, calcium channel–associated protein” (Myoscape), to play a key role in cardiac structure and function, including regulation of cardiac hypertrophy and contractility [12]. Myoscape deficiency in mice resulted in progressive heart failure characterized by myocardial hypertrophy, accompanied by a significant reduction in fractional shortening, the latter also occurring upon knockdown in zebrafish. Furthermore, a reactivation of fetal gene expression (Nppa, Nppb) was observed [12], as well as increased Calcineurin/NFAT signaling, both being associated with cardiac hypertrophy [13,14,15]. Moreover, Myoscape protein levels were found to be reduced by approximately 80% in myocardial tissue from patients with DCM compared to nonfailing human control hearts [12].

Striatin-interacting protein 1 (Strip1), a paralog of Myoscape, plays important roles in mammalian cells, regulating cellular signaling and actin cytoskeletal organization, affecting cell morphology and migration, but its function in the heart remains unexplored [16,17]. As a central adaptor of the STRIPAK complex, Strip1 connects STRIPAK components and ensures the positioning of PP2A near its targets, including Hippo kinases (MST1/2) and GCK III kinases (MST3/MST4), thereby playing a crucial role in their regulation [17,18,19,20,21]. MST1 and MST2 are the key kinases of the Hippo pathway, which serves as a critical regulator of organ size, cell proliferation, and tissue homeostasis [22]. Phosphorylation of MOB1 serves as a critical activation step for downstream Hippo signaling, as activation of the LATS–MOB1 complex ultimately leads to inactivation of the transcriptional co-activators YAP and TAZ, which serve as the downstream effectors [23]. Moreover, for several members of the Hippo pathway, including MST1 [24], involvement in pathological cardiac processes has already been described in detail. [23]. Since Strip1 regulates the aforementioned kinase activity in various non-cardiac cell types [6,17,18,20,21,22], we investigated whether Strip1 influences these cardiac-relevant STRIPAK components and Hippo pathway members in cardiomyocytes. We further assessed whether Strip1 serves a functional role in regulating pathological cardiomyocyte hypertrophy. This study addresses a key knowledge gap by exploring Strip1’s subcellular localization, its integration into cardiac STRIPAK, as well as Hippo and Calcineurin/NFAT regulation and its potential role as an anti-hypertrophic regulator.

## 2. Materials and Methods

### 2.1. Isolation and Culture of NRVCMs

NRVCMs were isolated from 1- to 2-day-old pooled male and female Wistar rats (Charles River Laboratories, Sulzfeld, Germany). Left ventricles were minced in ADS buffer (116 mM NaCl, 19.7 mM HEPES, 9.4 mM NaH_2_PO_4_∙H_2_O, 5.55 mM glucose, 5.36 mM KCl, 0.83 mM MgSO_4_; all from Sigma-Aldrich, Merck KGaA, Darmstadt, Germany) and digested with 1 mL/heart of ADS containing 0.5 mg/mL collagenase type II (Thermo Fisher Scientific GmbH, Karlsruhe, Germany) and 0.6 mg/mL pancreatin (Sigma-Aldrich) for 20 min at 37 °C.

Cell suspensions were filtered (70 µm), and digestion was terminated with newborn calf serum (Thermo Fisher Scientific; 1:5 dilution). The process was repeated 2–3 times. Cells were centrifuged (950 rpm, 15 min), and cardiomyocytes were enriched by Percoll density gradient (1500 g, 30 min; Cytiva, Freiburg, Germany).

Cells were seeded in DMEM (Thermo Fisher Scientific) containing 10% fetal bovine serum (Cat #10270), 100 U/mL penicillin, 100 µg/mL streptomycin, and 2 mM L-glutamine (Cat #10378-016; all from Thermo Fisher Scientific GmbH, Dreieich, Germany), and cultured at 37 °C with 5% CO_2_, which served as the standard condition for both NRVCMs and adult rat ventricular cardiomyocytes (ARVCMs). For additional hypertrophic response NRVCMs were treated with 100µM Phenylephrine or 10 µM Isoproterenol (Sigma-Aldrich Chemie GmbH, Steinheim, Germany, Deutschland Cat #P6126/Cat #I5627).

### 2.2. Isolation and Culture of ARVCMs

Adult cardiomyocytes were isolated from female six-week-old Wistar rats by retrograde perfusion using a Langendorff system, as previously described [10]. After heparin injection and excision, hearts were perfused via the aorta with isolation buffer (120.4 mM NaCl, 14.7 mM KCl, 0.6 mM KH_2_PO_4_, 0.6 mM NaH_2_PO_4_∙H_2_O, 1.2 mM MgSO_4_∙7H_2_O, 10 mM HEPES, 4.6 mM NaHCO_3_, 30 mM taurine, 10 mM 2,3-butanedione monoxime, 5.5 mM glucose; all from Sigma-Aldrich, Merck KGaA, Darmstadt, Germany), followed by enzymatic digestion with collagenase II (Worthington Biochemical Corp, Lakewood, US.) and 40 µM CaCl_2_.

Cells were dissociated in stop buffer (isolation buffer containing 1% BSA and 12.5 µM CaCl_2_; Sigma-Aldrich, Merck KGaA) and filtered through a 200 µm mesh. Calcium was reintroduced stepwise (100, 400, and 900 µM; 10 min each), and 100,000 ARVCMs were seeded per well on laminin-coated Nunc™ Lab-Tek™ chamber slides (Cat #154461, Thermo Fisher Scientific GmbH, Dreieich, Germany).

Cells were maintained in M199-based medium (supplemented with 10 mM creatine, 20 mM taurine, 1% BSA, 100 U/mL penicillin, 100 µg/mL streptomycin, and 2 mM L-glutamine; all from Thermo Fisher Scientific GmbH, Dreieich, Germany) and processed for immunofluorescence staining the following day.

### 2.3. Human Myocardial Samples

Human left ventricular myocardial samples were obtained from explanted hearts of patients suffering from end-stage heart failure (NYHA class IV) who underwent heart transplantation due to either DCM (*n* = 10) or ICM (*n* = 9). Control myocardial tissues (*n* = 6) originated from nonfailing donor hearts collected during standard post-transplant assessments, as previously described [10]. Detailed clinical parameters such as age, sex, comorbidities, or medication at the time of transplantation were not available for all samples and were therefore not included in the analysis. All procedures followed approval by the ethics committee of the University Medical Center Göttingen. Samples were immediately excised in the operating theater, rapidly frozen in liquid nitrogen, and stored at −80 °C until analysis.

### 2.4. Cloning and Generation of Adenovirus

The coding sequence of rat Strip1 was amplified from cardiac DNA using the GATEWAY Cloning platform, with attB-flanked primers (fwd: 5′-GGGGACAAGTTTGTACAAAAAAGCTGGCACCatgccatgagtaagggattcctttc-3′ and rev: 5′-GGGGACCACTTTGTACAAGAAAGCTGGGTCGCCagacaaggcgacagggaca-3′) and inserted into the pDONR221 entry vector (Thermo Fisher Scientific GmbH, Karlsruhe, Germany) via BP recombination using Gateway^®^ BP Clonase™ II Enzyme Mix (Cat #11789-020, Invitrogen™, Thermo Fisher Scientific). After verification by sequencing (Eurofins GSC Luxembourg S.A., Rollingergrund, Luxembourg), the insert was transferred to the pAd/CMV/V5-DEST™ vector (Cat #V493-20, Invitrogen™, Thermo Fisher Scientific) using Gateway^®^ LR Clonase™ II Enzyme Mix (Cat #11791-020, Invitrogen™, Thermo Fisher Scientific).

Recombinant adenoviruses (AdStrip1) were produced in HEK293A cells and harvested by three freeze–thaw cycles. Viral supernatants were cleared by centrifugation, aliquoted, and stored at −80 °C until use.

### 2.5. Overexpression of STRIP1 in NRVCMs

NRVCMs were infected 24 h post-isolation with AdStrip1 or control adenovirus (AdLacZ, Thermo Fisher Scientific) at a concentration of 50 infectious units (ifu) per cell. Infection was performed in DMEM containing penicillin/streptomycin and L-glutamine, but lacking fetal bovine serum. Virus-containing medium remained on the cells for 48 h before medium replacement. Cells were collected 72 h post-infection for downstream analysis.

### 2.6. siRNA-Mediated Knockdown of STRIP1 in NRVCMs

NRVCMs were transfected with either Silencer™ Select Strip1 siRNA (Thermo Fisher Scientific GmbH, Karlsruhe, Germany; Cat #4390771; sense: 5′-GCA UCA AGG UGA UCC GGA Att-3′, antisense: 5′-UUC CGG AUC ACC UUG AUG Ctg-3′) or Silencer™ Select Negative Control No. 1 siRNA (Cat #4390843, Thermo Fisher Scientific) using the TransMessenger^®^ Transfection Reagent (Cat #301525, Qiagen GmbH, Hilden, Germany).

Transfection complexes were prepared according to the manufacturer’s instructions. Briefly, 128 pmol siRNA were combined with Enhancer R and Buffer EC-R, followed by addition of TransMessenger reagent. The complexes were diluted in serum- and antibiotic-free DMEM/F-12 medium (Thermo Fisher Scientific) and applied to the cells. 3 h after transfection, the medium was replaced with serum-reduced culture medium (and again after 48 h) and harvested after 72 h.

Transfections were performed in triplicate wells per condition (Strip1 or negative control siRNA) across multiple plates for parallel downstream analyses.

### 2.7. Zebrafish Morpholino-Mediated STRIP1-Knockdown

Zebrafish (*Danio rerio*) were maintained and bred under standardized laboratory conditions as previously described [12]. All experimental procedures were approved by institutional authorities and performed in accordance with the Guide for the Care and Use of Laboratory Animals (NIH Publication No. 85-23).

250 µM morpholino-modified antisense oligonucleotides targeting the splice donor site of intron 2 of zebrafish *strip1* (5′-TAGCACATAAACCGACACCGTCCAT-3′) or a standard control morpholino (Gene Tools, Philomath, USA) were microinjected into fertilized eggs. To inhibit pigmentation, 0.003% 1-phenyl-2-thiourea (PTU; Cat #P7629, Sigma-Aldrich, Merck KGaA, Darmstadt, Germany) was added to the embryo medium.

Still images and videos were recorded at 48 and 72 h post-fertilization using a Zeiss MCU II stereomicroscope (Zeiss Microscopy, Oberkochen, Germany).

Cardiac function was quantified from bright-field video recordings using Fiji/ImageJ. Ventricular contractility was assessed by fractional shortening (FS) as previously described by Eden et al. [12]. Briefly, end-diastolic and end-systolic frames were identified based on maximal and minimal ventricular dimensions, respectively, and the ventricular short-axis diameter was measured at a consistent anatomical position. Fractional shortening was calculated as the relative change between end-diastolic and end-systolic ventricular diameter.

Pericardial effusion was quantified from a representative frame with clearly visible cardiac anatomy. The inner contour of the pericardial sac and outlining of the heart area were manually delineated, and a pericardial effusion index was calculated as the relative fraction of the pericardial cavity not occupied by the heart. All measurements were performed using identical anatomical landmarks and imaging conditions.

### 2.8. Transverse Aortic Constriction (TAC) in Mice

The TAC procedure was conducted on male C57BL/6 mice aged between 8 and 10 weeks (Charles-River Laboratories, Sulzfeld, Germany), as described in our previous publication [10]. In short, following lateral thoracotomy, a 6-0 Prolene suture was placed around the transverse aorta, tightening it against a 27-gauge needle to induce controlled stenosis. The thorax was then closed and the pneumothorax was resolved. Control (sham) animals underwent identical surgical steps without the actual ligation. This experiment was conducted following approval by the local ethics committee and in accordance with the regulations set forth by the Ministry for Energy Transition, Agriculture, Environment, and Rural Areas of Schleswig-Holstein.

### 2.9. Cyclic Biaxial Stretch in NRVCMs

Mechanical stretch was applied to NRVCMs using an established in vitro model, which has been shown to induce a hypertrophic response with upregulation of classical hypertrophy-associated gene expression markers [10].

Cells were seeded at a density of 1 × 10^7^ cells per well onto collagen type I–coated silicone elastomer-bottomed 6-well plates (Bioflex^®^ Collagen Type I Culture Plate, Cat #BF-3001C, Dunn Labortechnik GmbH, Asbach, Germany) in DMEM supplemented with 10% fetal bovine serum (Cat #10270) and 1× penicillin-streptomycin-glutamine (Cat #10378-016; all from Thermo Fisher Scientific, Dreieich, Germany). After 24 h of incubation, cells were washed with PBS and maintained in serum-free DMEM.

Biaxial cyclic stretch (118% total elongation, 1 Hz) was applied for 48 h using the Flexcell^®^ FX-6000™ Tension System with FlexSoft^®^ FX-6000™ V1.0 software (Flexcell International Corporation, Burlington, NC, USA). Unstretched cells cultured under identical conditions on collagen-coated Bioflex^®^ plates served as controls.

### 2.10. Protein Extraction and Immunoblotting

NRVCMs were lysed by three freeze–thaw cycles in RIPA buffer (1% NP-40, 1% sodium deoxycholate, 0.1% SDS, 150 mM NaCl, 10 mM sodium phosphate, 1 mM DTT, pH 7.2; all from Sigma-Aldrich, Merck KGaA, Darmstadt, Germany) supplemented with phosphatase inhibitors (Cat #P5726, #P0044) and protease inhibitors (Cat #04693132001, Roche Diagnostics GmbH, Mannheim, Germany). Lysates were cleared (14,000× *g*, 10 min, 4 °C), and protein concentration was determined using the DC Protein Assay (Cat #5000111, Bio-Rad Laboratories GmbH, München, Germany).

Mouse and human heart tissues were homogenized in RIPA buffer with ceramic beads (Cat #KT03961-1-003.2, Peqlab, VWR International GmbH, Darmstadt, Germany) and processed similarly. Proteins were separated on 10–12% Criterion™ XT Bis-Tris gels (Bio-Rad), transferred to PVDF membranes (Cat #IPVH00010, Merck Millipore, Burlington, MA, USA) by wet transfer, and blocked with 5% milk in TBS-T (Cat #70166, Sigma-Aldrich).

Membranes were incubated with primary antibodies (Table 1) overnight (4 °C), followed by HRP-conjugated secondary antibodies (Cat #111-035-144, Dianova GmbH, Hamburg, Germany; 1:10,000). Signal detection was performed using ECL Prime (Cat #RPN2232, Cytiva via VWR) and imaged via FluorChem Q (Biozym, Hessisch Oldendorf, Germany) or X-ray films (Cat #34089, Thermo Fisher Scientific). Densitometry was conducted with Fiji (ImageJ2, Version 2.3.0/1.53t) software [25,26], values were normalized to housekeeping or total protein as appropriate.

### 2.11. Co-Immunoprecipitation (Co-IP)

Co-IP from NRVCMs was performed using erythrocyte lysing buffer (50 mM HEPES, 250 mM NaCl, 5 mM EDTA, 1% NP-40; all from Sigma-Aldrich, Merck KGaA, Darmstadt, Germany) as previously described [10]. Briefly, 500 µg protein from clarified lysates were incubated overnight at 4 °C with STRIP1 antibody or control IgG (Table 1) at 1:250. Immune complexes were captured using Dynabeads Protein G (Cat #10004D, Thermo Fisher Scientific, Dreieich, Germany) for 4 h at 4 °C, washed five times, and eluted in Laemmli buffer at 95 °C for 5 min. After bead removal, supernatants were used for immunoblotting.

### 2.12. RNA Isolation, cDNA Synthesis and qPCR

Total RNA from NRVCMs was isolated using the Quick-RNA™ MiniPrep Kit (Cat #R1055, Zymo Research Europe GmbH, Freiburg, Germany) including on-column DNase I digestion. RNA concentration and purity (A260/A280) were assessed using a NanoDrop™ Lite Spectrophotometer (Thermo Fisher Scientific GmbH, Karlsruhe, Germany; Cat #ND-LITE-PR).

Reverse transcription of 500 ng RNA was performed with the iScript™ cDNA Synthesis Kit (Cat #1708891BUN, Bio-Rad Laboratories GmbH, Feldkirchen, Germany), and cDNA was diluted to 1 ng/µL. Quantitative PCR was conducted using the iTaq™ Universal SYBR^®^ Green Supermix (Cat #1725124, Bio-Rad) on a QuantStudio™ 5 Real-Time PCR System (Life Technologies Holdings Pte Ltd., Singapore). Primer sequences used are listed in Table 2. Expression levels were normalized to RPL32 and calculated by the 2^−ΔΔCt^ method. Control samples were set to a reference value of 1. All reactions were run in technical triplicates, including no-template controls; statistical analysis was based on biological triplicates.

### 2.13. Immunofluorescence Staining

For localization studies, 100,000 NRVCMs or ARVCMs per well were seeded on laminin-coated Nunc™ Lab-Tek™ chamber slides (Sigma-Aldrich Chemie GmbH, Steinheim, Germany; L2020-1MG; 154461, Thermo Fisher Scientific GmbH, Dreieich, Germany) and fixed after 16 h. For high-throughput quantification of NRVCM cell area, 20,000 cells per well were plated in 96-well plates (Thermo Fisher Scientific) and fixed after treatment.

Cells were washed three times with PBS and fixed using 4% paraformaldehyde in PBS (P6148, Sigma-Aldrich, Merck KGaA, Darmstadt, Germany). After washing, cells were permeabilized using 0.3% Triton™ X-100 in PBS (T8787, Sigma-Aldrich), followed by additional washes. Blocking was performed in 10% normal horse serum in PBS (Gibco™, Thermo Fisher Scientific GmbH) for 1 h at room temperature.

Primary antibodies (Table 1) were diluted in blocking buffer and incubated overnight at 4 °C in a humidified chamber. After washing, fluorophore-conjugated secondary antibodies (Thermo Fisher Scientific) were applied for 1 h at room temperature in the dark.

Cells were mounted on coverslips (0107032, Marienfeld Superior, Lauda-Königshofen, Germany) using Fluoromount™ containing DAPI (F4680, Sigma-Aldrich, Merck KGaA).

Images for cell area analysis were acquired using an In Cell Analyzer 2200 (GE Healthcare Life Sciences, Buckinghamshire, UK; 29-0278-86). For each experimental condition, multiple cells were analyzed per biological replicate. Confocal microscopy for colocalization studies was performed using a TCS SP8 X system (Leica Microsystems CMS GmbH, Wetzlar, Germany; 8110002344), and image channels were merged with Fiji (ImageJ2, Version 2.3.0/1.53t) software [25,26].

### 2.14. Protein Sequence Alignment

The amino acid sequences of human STRIP1 (UniProt ID: Q5VSL9) and STRIP2 (UniProt ID: Q9ULQ0) were retrieved from the UniProt database [27]. Pairwise alignment was performed using the EMBOSS Matcher tool V6.6.0 provided by the EMBL-EBI web server [28]. Default settings were used, and results were visualized to identify conserved regions.

### 2.15. Structure Prediction

Predicted quaternary structures of human STRIP1 (AlphaFold ID: AF-Q5VSL9-F1, version 4) and STRIP2 (AlphaFold ID: AF-Q9ULQ0-F1, version 4) were obtained from the AlphaFold Protein Structure Database (https://alphafold.ebi.ac.uk/, accessed on 24 May 2024). Structural features and domain organization were assessed for comparison.

### 2.16. Use of Generative Artificial Intelligence Tools

Generative artificial intelligence (GenAI) tools, including ChatGPT versions 4.0, 4o, o4-mini, 4.5 and 5, as well as Perplexity—which is based on models such as GPT-4o, Claude 3.7 Sonnet—were employed to assist in text generation and to support literature and source retrieval during manuscript preparation.

### 2.17. Statistical Analysis

All statistical analyses were performed using GraphPad Prism 9 (version 9.5.1; GraphPad Software, San Diego, CA, USA). Normality and homogeneity of variance were assessed where appropriate. Outliers were identified using the ROUT method with a Q value of 1%. *n* represents the number of biological replicates; for qPCR and immunoblot analyses, technical replicates were averaged prior to statistical analysis. For cardiomyocyte cell area measurements, the mean cell area of each biological replicate was calculated and used as the statistical unit for all comparisons. Individual cell measurements were used solely for visualization and were not treated as independent replicates. Comparisons between two groups were conducted using unpaired Student’s *t*-tests assuming equal standard deviations and normal distribution. DCM and ICM groups were compared to nonfailing controls by ordinary one-way ANOVA without matching or pairing. Dunnett’s multiple comparisons test was applied as the post hoc procedure. Unless otherwise stated, data are presented as mean ± SEM, and error bars in bar graphs represent the standard error of the mean (SEM).

## 3. Results

### 3.1. Strip1 Interacts with Key STRIPAK Components in Cardiomyocytes

**Figure 1:**
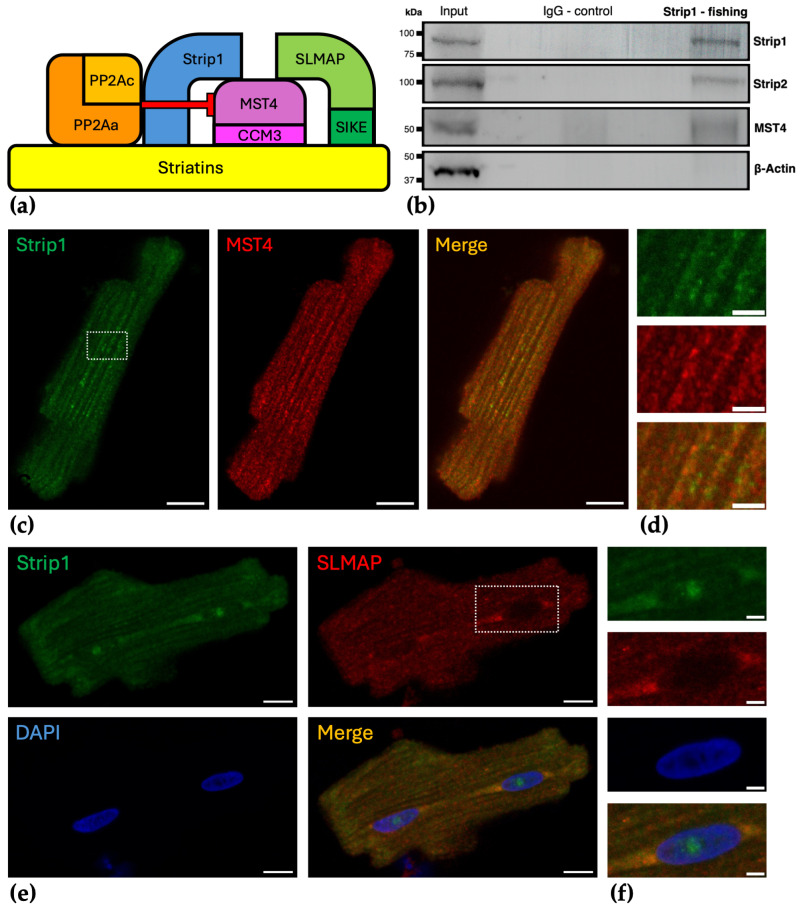
Strip1’s association with key cardiomyocyte STRIPAK components and its subcellular localization. (**a**) Proposed STRIPAK complex schematic in cardiomyocytes, based on diverse models from various contexts and cell types by Jeong et al. [17], Tang et al. 2019 [18] and Shi et al. 2016 [5], illustrating Strip1 centrally positioned with interactions to PP2A, striatins, and GCKII/GCKIII kinases, here represented by MST4 as a GCKIII kinase. Red T-shaped bar indicates inhibition of GCKII/GCKIII by PP2A [5,17,18]; (**b**) Co-IP from NRVCM lysates using Strip1 antibody for pulldown. Immunoblotting for Strip2 and MST4 detects bands in Strip1 pulldown samples, while β-actin serves as a negative control. Uncropped full-length blots are shown in Supplementary Figure S1; (**c**) Immunofluorescence staining of ARVCMs for Strip1 (green) and MST4 (red) demonstrates cytosolic colocalization in merged image; boxed area is enlarged in (**d**); (**e**) Immunofluorescence staining of ARVCMs for Strip1 (green) and SLMAP (red) with DAPI nuclear counterstain shows colocalization in the cytosol and perinuclear region; additional nuclear localization of Strip1 is observed; (**f**) boxed region from (**e**) enlarged; Scale bars: (**c**,**e**) 10 µm; (**d**,**f**) 3 µm;.

To investigate the role of Strip1 in cardiomyocyte signaling, we first examined its integration within the multi-enzyme STRIPAK complex in cardiomyocytes. Based on established models from other cell types (Jeong et al. 2021 [17]; Shi et al. 2016 [5]; Tang et al. 2019 [18]), Strip1 is proposed to function as a scaffold within the cardiac STRIPAK complex interacting directly with the phosphatase PP2A, MST4, and striatins, as illustrated in Figure 1a. The two-arm STRIPAK architecture is also presumed for the cardiac STRIPAK, with one arm involving Strip1 and the other SLMAP/SIKE1 anchoring GCKIII/GCKII kinases potentially regulated by PP2A [5,17,18]. Co-immunoprecipitation assays from NRVCM lysates showed that Strip1 was detected in association with key STRIPAK components Strip2 and the GCKIII kinase MST4 (Figure 1b), supporting its integral role within cardiac STRIPAK formation. Immunofluorescence microscopy of ARVCMs revealed Strip1’s cytosolic colocalization with MST4 (Figure 1c,d) as well as an overlapping localization with SLMAP in cytosolic and perinuclear regions (Figure 1e,f). Furthermore, a notable nuclear presence of Strip1 was observed (Figure 1e,f). These findings suggest a multifaceted subcellular distribution of Strip1, likely reflecting distinct functional roles.

### 3.2. Structurally Similar Strip1 and Strip2 Show Independent Regulation and Distinct Subcellular Localization in Cardiomyocytes

**Figure 2:**
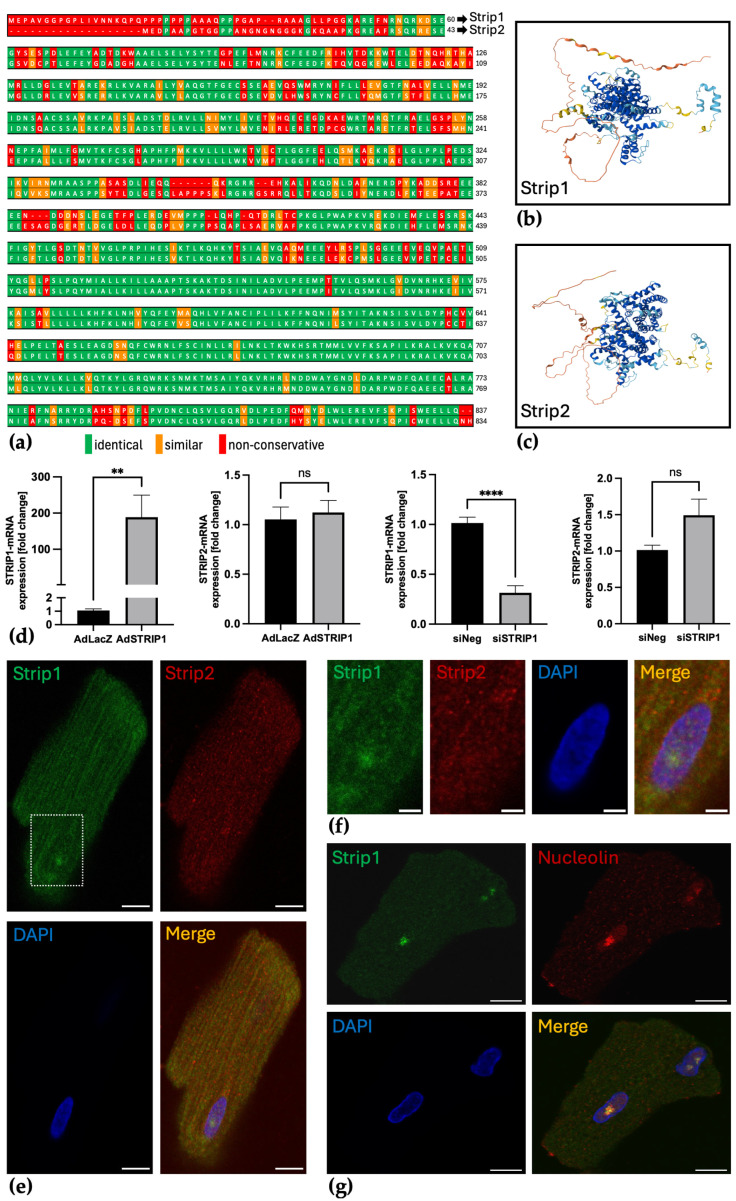
Structural similarity and distinct expression patterns of Strip1 and Strip2 in cardiomyocytes. (**a**) Alignment of Strip1 and Strip2 amino acid sequences using EMBOSS Matcher highlights conserved regions [28]; (**b**) Predicted structures of Strip1 and (**c**) Strip2 generated using AlphaFold [29,30] reveal similar domain organization. Color indicates per-residue model confidence (pLDDT): very high (pLDDT > 90, dark blue), high (70–90, light blue), low (50–70, yellow), and very low (<50, orange) [29,30]; (**d**) qPCR analysis of STRIP1 and STRIP2 mRNA following adenoviral-mediated overexpression of STRIP1 (AdSTRIP1, 50 ifu, 72 h—control: AdLacZ) or siRNA-mediated knockdown (siSTRIP1, 128 pmol, 96 h; control: siControl) in NRVCMs. While STRIP1 levels are significantly increased after AdSTRIP1 and decreased after siSTRIP1 compared to control, STRIP2 expression remains unchanged (*n* = 3); (**e**) Immunofluorescence staining of ARVCMs for Strip1 (green) and Strip2 (red) shows cytosolic and perinuclear colocalization; Strip1 additionally accumulates in an intranuclear round focus; (**f**) Enlarged view of boxed region from (**e**); (**g**) Immunofluorescence staining of ARVCMs for Strip1 and nucleolar marker Nucleolin demonstrates nucleolar colocalization; nuclei are counterstained with DAPI (blue); Scale bars: (**e**,**g**) 10 µm; (**f**) 3 µm; Student’s *t*-test; ns = not significant; ** *p* < 0.01; **** *p* < 0.0001; Data are shown as mean ± SEM.

Amino acid sequence alignment of human Strip1 and Strip2 (UniProt accessions Q5VSL9 and Q9ULQ0) performed with EMBOSS Matcher highlighted conserved regions, underscoring their structural similarity (Figure 2a). Further, predicted structures generated with AlphaFold confirmed analogous domain organization and confidence levels between the two proteins (Figure 2b,c). These similarities in primary and tertiary structures suggest that the paralogs may also share related functional roles. Functional modulation in NRVCMs using adenoviral overexpression and siRNA knockdown showed that STRIP1 levels can be specifically manipulated without altering STRIP2 expression at the mRNA level, indicating independent regulation (Figure 2d). Immunofluorescence in native ARVCMs showed that while both proteins colocalize in cytosolic and perinuclear regions, Strip1 uniquely localizes to a discrete intranuclear structure corresponding to the nucleolus, confirmed by co-staining with the nucleolar marker Nucleolin (Figure 2e–g). This distinct localization might indicate specialized nuclear functions for Strip1 not shared by Strip2.

### 3.3. Strip1 Expression Is Reduced in Human Cardiomyopathy and Under Mechanical Stress

**Figure 3:**
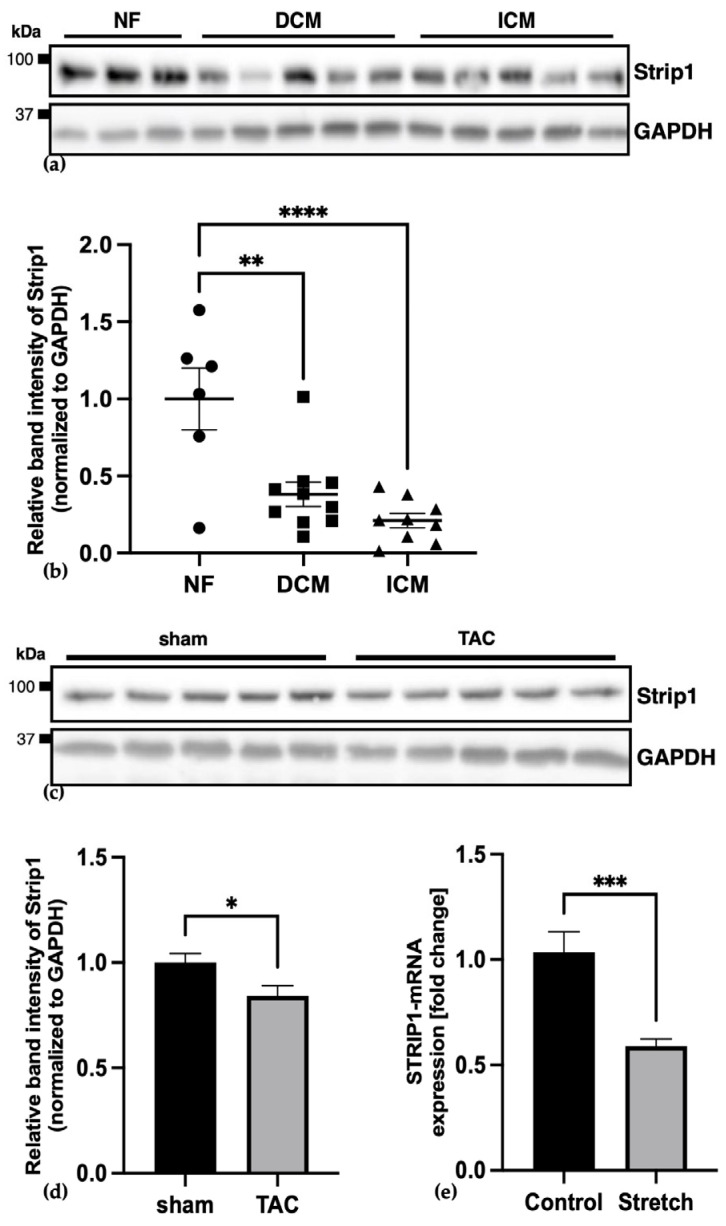
STRIP1 expression is reduced in human cardiomyopathy and models of mechanical stress. (**a**) Western blot analysis of Strip1 in human heart tissue from nonfailing (NF), DCM and ICM patients, with GAPDH as the loading control; (**b**) Densitometric quantification normalized to GAPDH shows significantly reduced STRIP1 expression in both DCM (*n* = 10 individuals) and ICM (*n* = 9) compared to nonfailing controls (*n* = 6); the representative blot does not show all biological replicates; (statistics: one-way ANOVA; * *p* < 0.05); (**c**) Western blot analysis of Strip1 in mouse heart samples reveals significantly decreased Strip1 protein levels following transverse aortic constriction (TAC) (*n* = 5) compared to sham-operated controls (*n* = 5), as quantified by (**d**) densitometry of the bands; (**e**) qPCR in NRVCMs after 48 h of mechanical stretch reveals significant downregulation of STRIP1 expression compared to unstretched control cells (*n* = 3); (**a**,**c**) Uncropped full-length blots are shown in Supplementary Figure S2; Student’s *t*-test; ns = not significant; * *p* < 0.05; ** *p* < 0.01; *** *p* < 0.001; **** *p* < 0.0001; Data are shown as mean ± SEM.

We next assessed Strip1 expression in human heart samples from patients with end-stage DCM or ICM, relative to nonfailing controls. Western blot analysis demonstrated a significant reduction of Strip1 protein in both DCM and ICM groups compared to nonfailing controls (Figure 3a,b). These clinical observations were mirrored in a murine model of pressure overload induced by transverse aortic constriction (TAC), where Strip1 protein levels significantly declined compared to sham-operated controls (Figure 3c,d), suggesting a mechanosensitive expression of STRIP1. Indeed, an in vitro mechanical stretch of NRVCMs showed downregulation of STRIP1 mRNA after 48 h, supporting the notion that mechanical stress directly influences STRIP1 expression (Figure 3e). Collectively, these data indicate that STRIP1 downregulation is a hallmark of pathological remodeling associated with heart failure and mechanical stress.

### 3.4. Strip1 Acts as a Negative Regulator of Cardiomyocyte Hypertrophy

**Figure 4:**
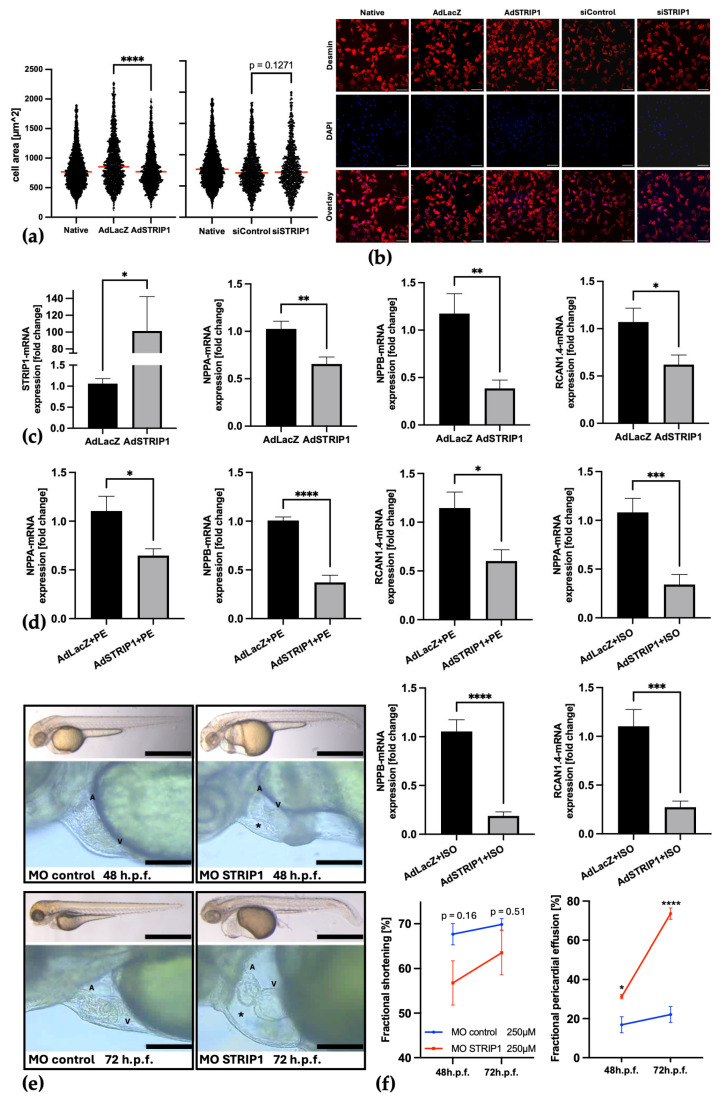
Strip1 modulates cardiomyocyte hypertrophy in vitro and in vivo. (**a**) Quantification of NRVCM area (µm^2^) under conditions: Native, siControl, siSTRIP1, AdLacZ and AdSTRIP1. Cell area in AdSTRIP1 is significantly reduced compared to AdLacZ, while NRVCMs after siSTRIP1 show no significant difference from siControl. Apart from changes in cell size, no obvious morphological alterations were observed. For visualization, the scatter plot displays individual cell measurements. Statistical analyses were performed using the mean cell area of each biological replicate as the statistical unit (*n* = 3 biological replicates); (**b**) Representative immunofluorescence images for each condition, showing Desmin (cell bodies), DAPI (nuclei), and merged overlays; High-resolution images of Figure 4b are shown in Supplementary Figure S4; (**c**) qPCR analysis shows that NPPA, NPPB, and RCAN1.4 mRNA expression is significantly reduced 72 h after AdSTRIP1 infection (50 ifu) compared to AdLacZ control in NRVCMs (*n* = 3); (**d**) with additional stimulation during the final 24 h with phenylephrine (PE, 100 µM or isoproterenol (ISO, 10 µM), this significant reduction in NPPA, NPPB and RCAN1.4 becomes more pronounced; (**e**,**f**) Zebrafish embryos with morpholino-induced STRIP1 knockdown (MO STRIP1; *n* = 5) at 48 hpf and 72 hpf (hours post fertilization) show reduced but non-significant fractional shortening compared to control morpholino-treated embryos (MO control; *n* = 4). In contrast, MO STRIP1 embryos exhibited significant pericardial effusion (=*), pronounced at 72 hpf (A = atrium, V = ventricle); Scale bars: (**b**) 100 µm; (**e**,**f**) 60 µm in whole-embryo image & 20 µm in cardiac close-up; Student’s *t*-test; ns = not significant; * *p* < 0.05; ** *p* < 0.01; *** *p* < 0.001; **** *p* < 0.0001; Data are shown as mean ± SEM.

Functional analyses in NRVCMs demonstrated that adenoviral-mediated overexpression of STRIP1 significantly reduced cardiomyocyte surface area relative to LacZ controls, indicating an antihypertrophic effect (Figure 4a). In contrast, STRIP1 knockdown via siRNA did not significantly alter cell size compared to control siRNA (Figure 4a,b). Compared with native NRVCMs, adenovirus-treated cells, including AdLacZ controls, displayed an increased cell size. No further obvious morphological alterations were observed apart from changes in cell size. At the molecular level, hypertrophic marker genes NPPA, NPPB, and RCAN1.4 were significantly downregulated following STRIP1 overexpression, suggesting that STRIP1 inhibits pathological hypertrophy (Figure 4c). This reduction was further pronounced when AdSTRIP1-infected NRVCMs were subjected to additional hypertrophic stimulation during the final 24 h using phenylephrine (PE) or isoproterenol (ISO) (Figure 4d), indicating that STRIP1 overexpression attenuates hypertrophic marker gene expression in response to adrenergic stress. Notably, the reduction in RCAN1.4, a known marker of calcineurin–NFAT pathway activity, suggests that Strip1 also suppresses this pro-hypertrophic signaling pathway (Figure 4c). In contrast, siRNA-mediated STRIP1 knockdown showed a trend toward increased expression of hypertrophic marker genes compared to control siRNA, both under basal conditions and following PE or ISO stimulation, although these changes did not reach statistical significance (Supplementary Figure S3). While fractional shortening was reduced in MO STRIP1 embryos compared to control morpholino-treated embryos, this difference did not reach statistical significance (Figure 4e,f). In contrast, pericardial effusion was significantly increased in MO STRIP1 embryos and was particularly pronounced at 72 h post fertilization, indicating compromised cardiac performance despite modest effects on systolic contractility (Figure 4e,f). No gross morphological abnormalities were observed in STRIP1 morphant zebrafish embryos compared to controls. Overall body morphology appeared comparable between groups. A tendency toward increased curvature of paravertebral skeletal muscle was noted in some morphants, but this was not further analyzed. Given the known limitations of morpholino-based approaches, including potential off-target effects, these in vivo findings are interpreted with caution. Accordingly, they provide supportive functional evidence for impaired cardiac performance following STRIP1 loss during development but are not intended as a standalone model of pathological hypertrophy. These results collectively underscore Strip1’s essential role in preventing pathological hypertrophy and maintaining cardiac function.

### 3.5. Strip1 Modulates Hippo Signaling and MST4 Kinase Activity

**Figure 5:**
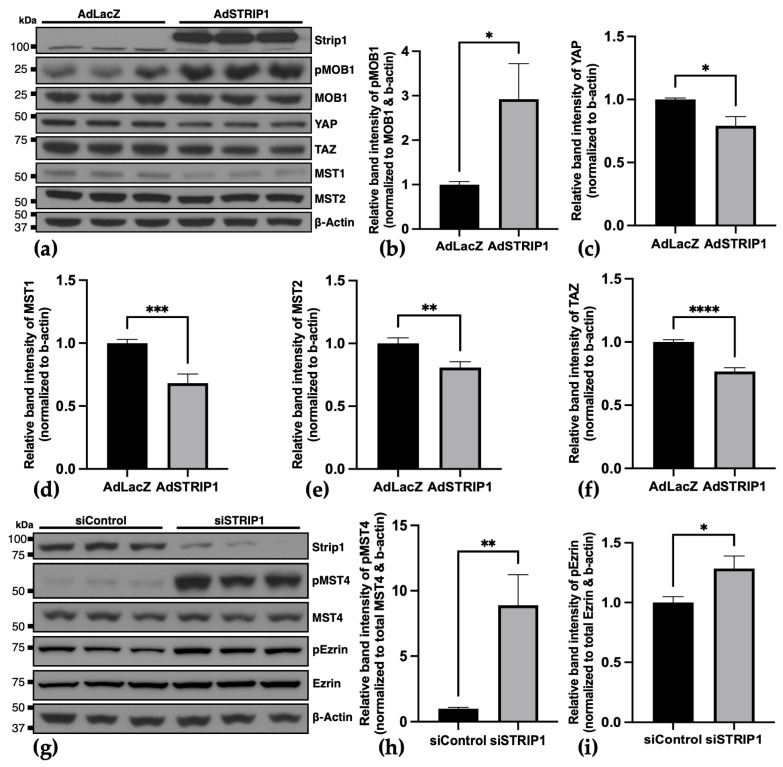
Strip1 modulates Hippo-pathway-related components and MST4 activity in NRVCMs. (**a**) Western blot analysis of Hippo pathway components in NRVCMs 72 h after infection with AdSTRIP1 (50 ifu) compared to AdLacZ control. (**b**–**f**) Densitometry of bands, normalized to β-actin and, for phosphorylated proteins, additionally to the respective total protein levels. Infection with 50 ifu AdSTRIP1 results in significantly increased (**b**) MOB1 phosphorylation, while total protein levels of (**c**–**f**) YAP, MST1, MST2 and TAZ are reduced significantly compared to control after 72 h. (**g**) Western blot analysis following STRIP1 knockdown (siSTRIP1) in NRVCMs shows a significant increase in phosphorylated MST4 (p-MST4), whereas total MST4 protein levels remain unchanged relative to control. Densitometric quantification of (**h**) p-MST4 relative to total MST4 and (**i**) relative to Ezrin, both normalized to β-actin. (**a**,**g**) Uncropped full-length blots are shown in Supplementary Figures S5 and S6; (**a**–**i**), *n* = 3; Student’s *t*-test; ns = not significant; * *p* < 0.05; ** *p* < 0.01; *** *p* < 0.001; **** *p* < 0.0001; Data are shown as mean ± SEM.

To explore downstream signaling pathways influenced by Strip1, we analyzed Hippo pathway–related signaling, previously implicated in STRIPAK signaling in non-myocytes [22,31]. In NRVCMs, adenoviral-mediated STRIP1 overexpression led to increased phosphorylation of MOB1, while total protein levels of YAP, MST1, MST2, and TAZ were significantly decreased relative to controls (Figure 5a–f). As these analyses were limited to total protein abundance for several components, these changes should not be interpreted as direct measures of pathway activity but rather suggest an association of cardiac Strip1 with Hippo-related signaling. Furthermore, STRIP1 knockdown elevated phosphorylated MST4 levels without altering total MST4 protein, providing direct evidence for altered MST4 kinase activation (Figure 5g–i), a GCKIII kinase and established STRIPAK component. Taken together, these results suggest that Strip1 is associated with the regulation of selected STRIPAK-related kinases, including Hippo pathway–associated components and the GCKIII kinase MST4, rather than establishing comprehensive regulation of GCKII kinases or Hippo pathway activity in cardiomyocytes.

## 4. Discussion

Our study identifies Strip1 as an essential negative regulator of pathological cardiomyocyte hypertrophy in vitro and in vivo and implicates Strip1 in cardiac STRIPAK-related signaling pathways. Notably, Strip1 overexpression attenuates hypertrophic marker gene induction not only under basal conditions but also in response to adrenergic stress, providing evidence for its function under pathophysiologically relevant stimulation. Importantly, Strip1 expression is markedly reduced in human dilated (DCM) and ischemic (ICM) cardiomyopathy, representing an association with advanced human heart failure and linking our experimental findings to clinically relevant disease states. This is the first evidence directly implicating Strip1 in cardiac biology and remodeling, thereby expanding our understanding of hypertrophic signaling and its translational significance for heart failure.

We recently confirmed the existence of a cardiac STRIPAK complex while investigating the role of MST4 [10]. Co-IP and immunofluorescence analyses showing Strip1 interaction and colocalization with Strip2/Myoscape, MST4, and SLMAP in cardiomyocytes now also support the involvement of Strip1 in cardiac STRIPAK-associated complexes. Strip1 may serve a core scaffolding role, as established in other cell types [6]. The perinuclear and cytoplasmic colocalization of Strip1 and Strip2 further suggests functional cooperation in cardiomyocytes, consistent with STRIPAK organization in other systems [5].

Strip1 and Strip2 share structural similarity [32] and morpholino-mediated Strip1 knockdown in zebrafish displays impaired cardiac function characterized by significant pericardial effusion and a trend toward reduced contractile performance, similar to Myoscape-deficient zebrafish, which showed severely impaired ventricular contractility and the development of pericardial effusion [12]. Nevertheless, their functions are non-redundant: Strip1 knockout causes embryonic lethality due to impaired mesodermal migration [16], while Strip2-deficient mice survive but develop severe heart failure [12]. The absence of Strip2 compensation following Strip1 knockdown underlines this specificity.

The perinuclear localization of Strip1 in cardiomyocytes resembles STRIPAK component patterns in other cell types, likely reflecting the Golgi or, in the case of SLMAP, the nuclear envelope association [6,33]. However, the apparent accumulation of Strip1 in the nucleolus of ARVCMs is unexpected. In murine podocytes, Strip1 is nuclear but homogeneously distributed throughout the nucleoplasm [34], whereas nucleolar localization has only been described for Strip2 in murine embryonic CGR8 stem cells, alongside its cytosolic and perinuclear distribution [35].

While the nucleolus is primarily known for ribosome production [36], cell cycle regulation and apoptosis [37], a connection between its function and cardiac disease has also been established [38]. For instance, nucleolar enlargement associated with increased ribosomal activity has been observed in human ICM and DCM [39]. Additionally, nucleolar organizer region (NOR) activity correlates positively with cardiac mass and left ventricular hypertrophy in patients with hypertension but is reduced in ICM and heart failure [40], as reviewed by Hariharan et al. [38].

Recently, specific nucleolar proteins, including Nucleolin, Nucleophosmin, and Nucleostemin, have been uncovered to influence cardiomyocyte growth, survival and stress adaptation [38,41,42]. Our immunofluorescence-based observation suggesting a nucleolar association of Strip1 raises the question whether Strip1 joins this growing list of nucleolar proteins, integrating stress signals and growth responses in the heart.

Cardiac hypertrophy, a key contributing factor in the development of cardiomyopathies and heart failure [43], appears to be negatively regulated by Strip1, as indicated by multiple experimental approaches. Strip1 overexpression reduces cardiomyocyte size and expression of hypertrophic markers NPPA and NPPB under basal conditions and further attenuates adrenergic stress–induced expression of NPPA, NPPB, and RCAN1.4 following phenylephrine or isoproterenol stimulation, while knockdown increases expression of Nppa. In contrast, loss-of-function approaches yielded only modest and variable molecular responses, suggesting that reduced Strip1 levels alone are not sufficient to robustly drive hypertrophic gene activation in this setting (Supplementary Figure S1). These in vitro findings are complemented by the zebrafish data, which provide supportive in vivo evidence for compromised cardiac performance following STRIP1 loss rather than a standalone model of pathological hypertrophy.

Therefore, Strip1’s downregulation in ICM may reflect a modest and potentially compensatory response, as myocardial hypertrophy is a known initially adaptive response to the functional insufficiency of damaged myocardial tissue under ischemic conditions [44]. Comparable mechanisms of compensatory remodeling may also account for Strip1’s downregulation in DCM, an observation consistent with prior reports of decreased Strip2 expression in end-stage DCM patients [12]. Given the relatively small but statistically significant changes observed, this interpretation should be viewed cautiously. Strip1 expression also declines under mechanical stress—after TAC and in vitro stretch —further supporting its role in modulating hypertrophic adaptation under pathological load.

We observed Strip1’s influence on multiple hypertrophy-regulating signaling pathways, likely underlying its anti-hypertrophic effects. One key pathway is Calcineurin/NFAT, a well-characterized mediator of pathological cardiac hypertrophy [45]. Calcineurin activates NFAT, promoting hypertrophic gene expression [46], with sustained activation leading to DCM and heart failure in mouse models [46]. Moreover, in patients with DCM and NYHA class IV heart failure, calcineurin activity is elevated by approximately 80% compared to nonfailing hearts, along with increased activation of NFAT transcription factors [47]. Notably, the calcineurin–NFAT pathway is activated in pressure overload models of pathological hypertrophy but not in endurance training models of physiological hypertrophy [48]. Strip1 overexpression significantly reduces RCAN1.4 expression, which is widely regarded as a robust and sensitive readout of Calcineurin/NFAT pathway activity due to the high density of NFAT binding sites within its promoter region [49]. As RCAN1.4 is induced almost exclusively upon Calcineurin activation in cardiomyocytes, its downregulation both under basal conditions and in response to adrenergic stimulation suggests that Strip1 limits Calcineurin/NFAT-driven pro-hypertrophic signaling [49]. This is consistent with findings in STRIP2 knockout mice, where excessive activation of the calcineurin/NFAT pathway was observed following TAC. [12] Since enhanced Calcineurin activity occurs in both Strip1- and Strip2-deficient conditions, both paralogs likely contribute to suppressing pathological hypertrophy, at least in part via this pathway [12].

Another mechanism may involve Strip1’s association with Hippo signaling pathway, which governs heart size via regulation of proliferation during development and hypertrophy in adult cardiomyocytes [23,50]. For one, Strip1 overexpression reduced YAP expression, as assessed at the total protein level. In patients with hypertrophic cardiomyopathy (HCM), YAP activity and gene expression are elevated [51], consistent with findings in NRVCMs where YAP1 overexpression promotes and knockdown attenuates hypertrophy [52].

In transgenic mice, MST1 overexpression induces apoptosis and DCM, while it fails to elicit compensatory hypertrophy in the surviving cardiomyocytes [24]. Yet its expression is reduced upon Strip1 overexpression, which appears counterintuitive. However, only total MST1 expression was assessed, while phosphorylation status, indicating its activation, was not analyzed. Changes in total protein abundance may reflect altered transcription, protein stability, or cellular stress responses rather than pathway activity per se. The increased MOB1 phosphorylation observed upon Strip1 overexpression in NRVCM is further compatible with altered Hippo signaling, in line with findings in HEK293A cells [17]. Notably, STRIP1 knockout in HEK293A cells results in increased MST1 phosphorylation. [22]. Although the effects of Strip1 on Hippo pathway components are not unidirectional, these data collectively suggest a potential association between Strip1 and Hippo-related signaling in cardiomyocytes but should be interpreted cautiously and do not constitute definitive evidence for direct regulation of Hippo pathway activity.

Enhanced phosphorylation of MST4 following STRIP1 knockdown is also consistent with prior reports in other cell types, where Strip1 contributes to MST4 inhibition within the STRIPAK complex [21]. As illustrated in Figure 1a, adapted from established STRIPAK models [5,18], Strip1 likely forms a scaffold with striatins in cardiomyocytes to facilitate recruitment of the phosphatase PP2A to GCKIII kinases such as MST4 [6]. The observed increase in Ezrin phosphorylation at Thr567 upon STRIP1 knockdown in NRVCMs is most likely attributable to the elevated activation of MST4. Previous studies in W4 cells demonstrated MST4-dependent Ezrin phosphorylation at this site [11], whereas direct interaction between Strip1 and Ezrin was not seen in large-scale interactome studies using JEG-3 epithelial cells [53]. Together, these findings suggest that Strip1 not only modulates MST4 activity in NRVCMs but subsequently modulates its effects on downstream substrates. Since MST4 overexpression promotes hypertrophy in NRVCMs [10], while Strip1 has the opposite effect, their inverse expression patterns under mechanical stress and in DCM samples [10] may suggest that Strip1 exerts anti-hypertrophic effects, at least in part by limiting MST4-associated signaling within the STRIPAK complex. In contrast, MST4 is markedly upregulated in samples of DCM and ICM [10]. Similarly, following in vitro biaxial stretch, MST4 expression is upregulated along with stress biomarkers NPPA and NPPB [10], while Strip1 expression is decreased. These reciprocal expression patterns may suggest opposing roles for Strip1 and MST4 in cardiomyocytes and their contribution to cardiac pathology.

A recently identified chromatin-associated complex in cardiomyocytes—comprising Calcineurin, its inhibitor Carabin, MST3, and the histone monomethyltransferase MLL3—has been shown to epigenetically regulate cardiac hypertrophy [54]. Under basal conditions, Carabin inhibits Calcineurin, while MST3 suppresses MLL3 activity via phosphorylation. Upon mechanical stress, induced by TAC, displacement of Carabin and MST3 allows subsequent MLL3 and Calcineurin activation, both promoting hypertrophic gene expression [54]. Our observations that Strip1 affects not only Calcineurin activity but also MST4 phosphorylation, a GCKIII kinase closely related to MST3, suggest that both pathways may converge downstream on MLL3, which regulates cardiac hypertrophy and, like Strip1, is localized to the nucleus.

However, a potential discrepancy arises in that MST3 appears to act as a brake on MLL3-mediated hypertrophy within the complex [54], while our previous data on MST4 [10] suggest a prohypertrophic influence of GCKIII kinases. Together with the observed increase in MST4 activity following STRIP1 knockdown, this raises important questions about the precise role of MST3/4 activity in different cellular contexts and underscores the need for further investigation into how Strip1 interacts with these kinases and the chromatin regulatory machinery [54].

Future investigations should explore the role of Strip1 in cardiomyocyte apoptosis, a promising avenue given the known involvement of nucleolar proteins in apoptotic signaling, as well as the pro-apoptotic roles of the kinases MST1, MST2, and MST4, which we showed to be influenced by Strip1 in NRVCMs. We previously observed reduced cleavage of Caspase-3 and -7 in NRVCMs upon MST4 overexpression, indicating suppression of apoptosis. Conversely, MST4 knockdown significantly increased Caspase-7 activity [10]. Cardiac-specific overexpression of MST1 in transgenic mice has been associated with increased caspase activity and cardiomyocyte apoptosis [24], whereas MST2-deficient mice exhibit reduced apoptosis [55]. Depending on cellular context, however, MST1 and MST2 may also exert anti-apoptotic effects [56,57]. The apparent contradiction, putative pro-apoptotic influence of Strip1 via MST4 regulation versus potentially anti-apoptotic effects via MST1/MST2 downregulation, suggests complex and potentially compensatory mechanisms that warrant further elucidation.

Limitations: Although our data suggest a role for Strip1 in regulating cardiac hypertrophy, a murine knockout model will be required for further validation. As global deletion of STRIP1 is lethal [16], a heart-specific knockout using αMHC-Cre recombination is planned for future studies. The use of morpholino-mediated STRIP1 knockdown in zebrafish represents a limitation, as morpholinos can be associated with off-target or non-specific effects. Therefore, the zebrafish data were used not as a primary basis for mechanistic interpretation, but rather to provide supportive in vivo evidence in line with the in vitro findings. Although no overt morphological defects were observed in STRIP1 morphants, additional uncharacterized structural or functional abnormalities cannot be excluded. Furthermore, mechanistic analyses were largely limited to total protein expression, as phosphorylation status, subcellular localization, and downstream transcriptional activity—particularly TEAD-dependent output of the Hippo pathway—were not directly assessed. Accordingly, changes in YAP/TAZ abundance are interpreted as suggestive rather than definitive evidence of Hippo pathway modulation. The functional significance of Strip1’s putative nucleolar localization remains to be determined, particularly its involvement in ribosome biogenesis, stress responses, or apoptosis, and will require biochemical validation in future studies.

## 5. Conclusions

In summary, our findings establish Strip1 as a novel and central regulator of pathological cardiac hypertrophy, whose expression is significantly reduced in human DCM and ICM and under mechanical stress—highlighting its potential as a key protective factor during cardiac disease. The observed nucleolar association of Strip1 suggests a previously unrecognized nuclear aspect of its function in cardiomyocytes. Mechanistically, Strip1 limits hypertrophic remodeling in response to adrenergic stress by influencing pro-hypertrophic signaling pathways, including the STRIPAK-associated kinase MST4, as well as Calcineurin/NFAT- and Hippo-related signaling. These findings extend our understanding of cardiac STRIPAK-related functions and suggest Strip1 as a potential regulator and target of pathological cardiac remodeling.

## Figures and Tables

**Table 1:** Primary antibodies used for Immunoblotting and Immunofluorescence.

Target	Supplier	Catalog No.	Host Species	Dilution
STRIP1 (OTI7B8)	Novus Biologicals, Centennial, CO, USA	NBP2-45715	Mouse	1:5000 (WB), 1:250 (Co-IP), 1:100 (IF)
MST4 (EP1864Y)	Abcam PLC, Cambridge, UK	ab52491	Rabbit	1:150,000–1:100,000 (WB), 1:500 (IF)
FAM40B	Proteintech, Planegg, Germany	25163-1-AP	Rabbit	1:10,000–1:1000 (WB), 1:100 (IF)
Phospho-MST3/4/STK25 (T174/178/190)	Abcam PLC, Cambridge, UK	ab76579	Rabbit	1:1300 (WB)
Phospho-Ezrin (Thr567)	BioVision, Milpitas, CA, USA	A-1970	Rabbit	1:500–1:1000 (WB)
Ezrin	Novus Biologicals, Centennial, CO, US	NBP2-16396	Rabbit	1:10,000 (WB)
Phospho-YAP (Ser397) LATS1 Phospho-MOB1 (Thr35) MOB1 MST1 MST2 SAV1 Phospho-YAP (Ser127) YAP/TAZ	Cell Signaling Technology Europe B.V., Leiden, The Netherlands	13619 3477 8699 13730 3682 3952 13301 13008 8418	Rabbit	1:1000 (WB)
β-Actin	Santa Cruz Biotechnology, Heidelberg, Germany	sc-47778	Mouse	1:50,000 (WB)
Desmin	Abcam PLC, Cambridge, UK	ab15200	Rabbit	1:700 (IF)
SLMAP	Novus Biologicals, Centennial, CO, USA	NBP1-81398	Rabbit	1:1000 (IF)
Nucleolin	Novus Biologicals, Centennial, CO, USA	NB600-241	Rabbit	1:50 (IF)
Normal mouse IgG control	Sigma Aldrich, Taufkirchen, Germany	12-371	Mouse	1:250 (Co-IP)
Adenovirus Type 5 Hexon (FITC)	Thermo Scientific, Merelbeke, Belgium	PA1-73053	Goat	1:50 (IF)

**Table 2:** Primers used for qRT-PCR.

Target	Forward Sequence	Reverse Sequence
STRIP1 rat	ACG AGC TTC CAG AGC TAA CC	ACA CCA CCA GCA TCA TAG TCC
STRIP1 mouse	CTT CCG GAT CCA TGT GTC AGA	AGC TGC ACT CTC CAA AGG TA
STRIP2 rat	CTT CCG AAC TGA ACT GAG TTT CT	CAA ATC CAC CAA GGG TAA ACA
STRIP2 mouse	CCA GGA CCA TGA TGT TAG TGG TG	GCT TAT CAT ATC GGC GGC TG
RPL32 rat/mouse	GGT GGC TGC CAT CTG TTT TAC G	CCG CAC CCT GTT GTC AAT GC
NPPA rat/mouse	GGA GCA AAT CCT GTG TAC AGT G	ACC TCA TCT TCT ACC GGC AT
NPPB rat/mouse	GCA GCA TGG ATC TCC AGA AGG	CTG CAG CCA GGA GGT CTT CC
RCAN1.4 rat/mouse	TAG CTC CCT GAT TGC TTG TG	GGA TTC AAA TTT GGC CCT GG

## Data Availability

Data underlying this study are available within the article and its Supplementary Materials. The raw image datasets from cell size area measurements (approximately 120 GB) and further information are available from the corresponding author upon reasonable request.

## Supplementary Materials

The following supporting information can be downloaded at: http://bit.ly/40GKzBF (accessed on 2 March 2026), Figure S1: Strip1 CoIP Western blots; Figure S2: TAC/mice and human DCM and ICM Western blots; Figure S3: Strip1 knockdown catecholamine stimulation; Figure S4: High-resolution images of cell area measurement; Figure S5: Strip1 overexpression Hippo members Western blots; Figure S6: Strip1 knockdown p-MST4/Ezrin Western blots.

## Abbreviations

The following abbreviations are used in this manuscript:

AdLacZ	Adenoviral LacZ construct
AdSTRIP1	Adenoviral STRIP1 construct
αMHC-Cre	Alpha-myosin heavy chain–driven Cre recombinase
ARVCMs	Adult ventricular cardiomyocytes
BSA	Bovine serum albumin
Co-IP	Co-immunoprecipitation
DCM	Dilated cardiomyopathy
DMEM	Dulbecco’s Modified Eagle Medium
DTT	Dithiothreitol
GAPDH	Glyceraldehyde-3-phosphate dehydrogenase
GCKII/GCKIII	Germinal Center Kinase class II/class III
HCM	Hypertrophic cardiomyopathy
HEPES	4-(2-hydroxyethyl)-1-piperazineethanesulfonic acid
hpf	Hours post fertilization
ICM	Ischemic cardiomyopathy
ifu	Infectious units
ISO	isoproterenol
MLL3	Histone H3K4 mono-methyltransferase MLL3 (KMT2C)
MO STRIP1	morpholino-induced STRIP1 knockdown
MOB1	Mps one binder 1
MST	Mammalian sterile 20-like kinase
Myoscape	Myocardium-enriched, calcium channel–associated protein
NFAT	Nuclear Factor of Activated T-Cells
NOR	Nucleolar organizer region
NPM	Nucleophosmin
NPPA	Natriuretic peptide A gene
NPPB	Natriuretic peptide B gene
NRVCMs	Neonatal rat ventricular cardiomyocytes
NYHA	New York Heart Association functional class
PBS	Phosphate-buffered saline
PE	phenylephrine
PP2A	Protein phosphatase 2A
PTU	1-Phenyl-2-thiourea
RCAN1.4	Regulator of Calcineurin 1 isoform 4
RIPA buffer	Radioimmunoprecipitation assay buffer
RPL32	Ribosomal protein L32 (Housekeeping gene)
SDS	Sodium dodecyl sulfate
SIKE1	Suppressor of IKKε 1
siRNA	Small interfering RNA
siSTRIP1/siNeg	siRNA targeting STRIP1 mRNA/Negative Control
SLMAP	Sarcolemmal membrane-associated protein
TAC	Transverse aortic constriction
TAZ	Transcriptional co-activator with PDZ-binding motif (WWTR1)
UniProt	Universal Protein Resource
YAP	Yes-associated protein
