# Supplementary figures and images for: Strip1 Is a Novel Negative Regulator of Cardiomyocyte Hypertrophy
Source: Cells. 2026 Mar 18;15(6):540. doi: 10.3390/cells15060540 (PMC13025875; doi:10.3390/cells15060540)

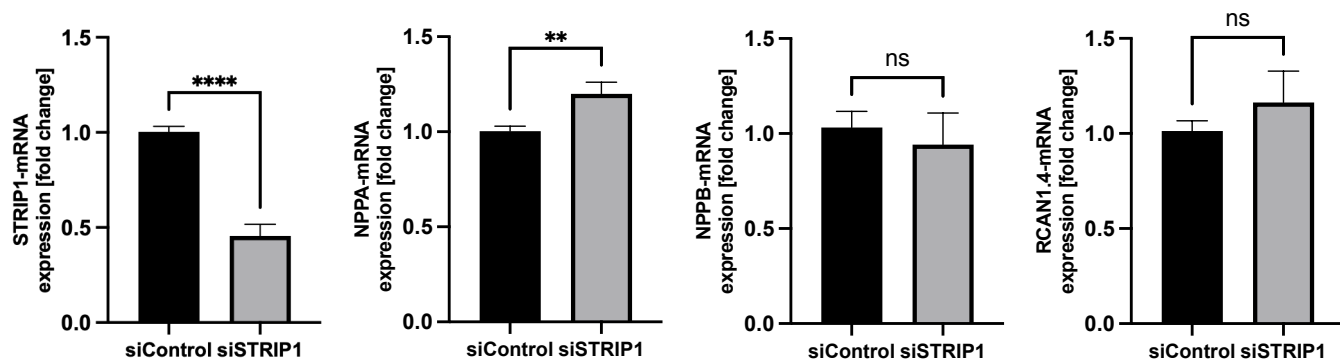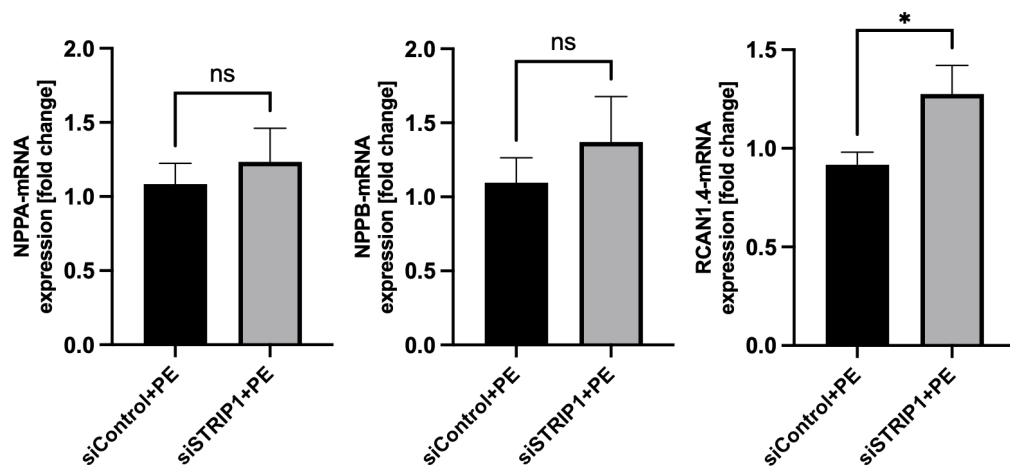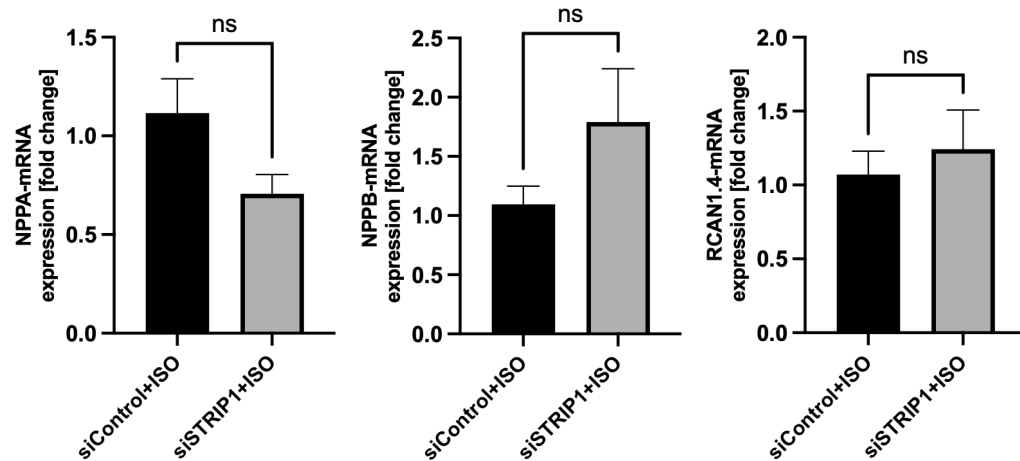

Supplement: Supplementary file 1 [file cells-15-00540-s001.zip › cells-4049057-supplementary.pdf]
